# Food Hygiene Surveillance in Italy: Is Food Ice a Public Health Risk?

**DOI:** 10.3390/ijerph17072408

**Published:** 2020-04-02

**Authors:** Giuseppina Caggiano, Vincenzo Marcotrigiano, Paolo Trerotoli, Giusy Diella, Serafina Rutigliano, Francesca Apollonio, Angelo Marzella, Francesco Triggiano, Matilde Gramegna, Domenico Lagravinese, Giovanni Trifone Sorrenti, Pantaleo Magarelli, Umberto Moscato, Maria Teresa Montagna

**Affiliations:** 1 Department of Biomedical Science and Human Oncology Hygiene Section–University of Bari Aldo Moro, 70124 Bari, Italy; paolo.trerotoli@uniba.it (P.T.); giusy.diella@uniba.it (G.D.); rutigliano.s@gmail.com (S.R.); francesca.apo@libero.it (F.A.); marzella.angelo@libero.it (A.M.); francesco.triggiano@uniba.it (F.T.);; 2 Department of Prevention, Food Hygiene and Nutrition Service, Local Health Unit BT, Barletta-Andria-Trani, 76125 Trani, Italy; vincenzo.marcotrigiano@aslbat.it (V.M.); sorrentigianni@tiscali.it (G.T.S.); pantaleo.magarelli@aslbat.it (P.M.); 3 Department of Prevention, Food Hygiene and Nutrition Service, Local Health Unit Bari–Metropolitan Area, 70100 Bari, Italy; matilde.gramegna@asl.bari.it; 4 Department of Prevention, ASL Bari, 70132 Bari, Italy; dipartimento.prevenzione@asl.bari.it; 5 Department of Health Science of Woman and Child and Public Health-Occupational Health and Hygiene Section-Fondazione Policlinico Universitario “A.Gemelli” IRCCS, 00168 Rome, Italy; umberto.moscato@unicatt.it

**Keywords:** food ice, ice machines, Public Health, microbial contamination, *E. coli*, coliform, food hygiene, fungi

## Abstract

Food ice is used as an ingredient or as a coolant in drinks and in the storage of food, especially fishery products. Studies show that ice can be polluted both by chemical substances and by bacteria and fungi. In particular, the presence of fungi in these food matrices has acquired an important role in Public Health, as it can represent a risk factor for fungal complications in immunocompromised subjects. In the present study we evaluated the hygiene–sanitary quality of food ice from public and collective catering establishments in a large area of Southern Italy, investigating the mandatory parameters (*Escherichia coli,* coliform and Enterococci) and some accessory parameters (*Staphylococcus aureus, Pseudomonas aeruginosa* and fungi) provided for Italian Legislative Decree 31/01. Although 54.5% of samples were compliant, the results highlight a vast contamination of food ice by bacteria and fungi. In particular, 95.8% of samples were contaminated by fungi, stressing no difference between compliant and non-compliant samples. Their presence is generally attributable to the poor sanitation conditions in the production and/or administration phase and to the incorrect sanitization and ordinary maintenance procedures. It seems appropriate to suggest the need to carry out a specific risk assessment with respect to the self-control plans.

## 1. Introduction

Worldwide food ice production has increased in recent years because of the high demand for ice cubes in public places such as bars, pubs and restaurants. Today, commercial ice production is estimated at around 500,000 tonnes per year in Europe and 5,600,000 tonnes per year in the United States [1]. Used in large quantities mostly in the summer, ice is useful in restorative and commercial activities for preparing cold drinks, and helps to maintain the cold chain that is very important for multiple foods, including fish and other foods eaten raw.

Among European countries, Spain has the highest ice consumption at over 400,000 tonnes per year, of which 50% is self-produced, whereas the other 50% is produced and packaged in production plants [2]. In Italy, ice consumption is estimated at 58,000 tonnes in daytime bars and 25,000 tons in restaurants each year, of which 60% is consumed between June and September and consumption is expected to exceed 500,000 tonnes per year in the coming years [2].

Although numerous benefits are conferred by the use of food ice, the scientific literature shows how it can be contaminated by different chemical and physical substances [3,4]. In fact, toxic inorganic substances such as arsenic-type heavy metals, chromium, mercury, cadmium and lead, which are often found in industrial wastewaters or arising from the use of phyto-drugs, can penetrate the soil and pollute both superficial and deep waters. Even when "Food ice" is defined as “ice prepared with drinking water, brought to a temperature equal to or lower than 0 °C, and when melted, the water will have the same requisites foreseen in the Italian Legislative Decree 2 February 2001, n. 31” [5], it can represent a vehicle for the transmission of pathogenic agents associated with foodborne illnesses.

Many studies have investigated the microbiological quality of food ice and have confirmed its potential role as a vehicle for foodborne illnesses. In fact, Enterobacteriaceae, Enterococci and fungi have been isolated from ice in Italy [1,6,7]; total and faecal coliforms, *Clostridium perfringens, Pseudomonas aeruginosa, Yersinia* spp. and *Salmonella* spp. in Greece [8,9]; *Salmonella Saintpaul* in Australia [10]; multi-antibiotic-resistant strains such as *Escherichia coli ESLB* (extended-spectrum-beta-lactamase) in Vietnam; and some episodes of cholera related to *Vibrio cholerae O1 Ogawa ctxA* have been linked with ice melted in tea [11,12].

The yeast and mould contamination of food ice has also acquired an important role in Public Health, as it is considered among the risk factors for fungal complications in immunocompromised subjects [6].

Finally, ice produced from contaminated water caused an epidemic of a Norwalk-like virus in Puerto Rico with approximately 5000 cases [13].

With this background in mind, the aim of the present study was to investigate the hygiene–sanitary quality of food ice from public and collective catering establishments in a large area of Southern Italy, and to assess whether these establishments complied with Decree No. 31/2001 [5] on food ice.

## 2. Materials and Methods

### 2.1. Ice Cube Collection

Ninety-nine ice samples were taken directly from the storage compartments of ice machines located in public and collective catering establishments in an urban geographic context. Each sample equal to 1 kg of ice in cubes was placed in sterile food bags and transported to our laboratory in a special isothermal fridge at a controlled temperature of 4 °C to be analysed within 24 h of collection.

All ice samples were taken in public and collective catering establishments during the normal operation of the food business activity from active ice machines connected to a public water supply network.

### 2.2. Determining Contamination Levels in Ice Samples

After melting at room temperature, the ice samples were evaluated for the mandatory parameters provided for in Decree No. 31/2001 relating to *E. coli,* coliforms and Enterococci, and some accessory parameters relating to *Staphylococcus aureus, P. aeruginosa* and fungi. Each sample was filtered through a cellulose ester membrane with a diameter of 47 mm and a pore size of 0.45 μm (Millipore, Milan, Italy). The samples were considered compliant when *E. coli*, coliforms and Enterococci were absent in 100 mL of each melted ice sample.

### 2.3. E. coli and Coliform Investigation

A 100 mL aliquot of melted ice was filtered and the membrane placed on plates containing Chromogenic Coliform Agar (Biolife Italiana Srl, Milan, Italy). After incubation at 36 ± 2 °C for 24 ± 2 h, the blue-violet colonies that formed were identified as *E. coli*, and the salmon pink, oxidase negative ones were identified as coliforms [14].

### 2.4. Enterococci Investigation

A 100 mL aliquot of melted ice was filtered, the membrane was placed on Slanetz and Bartley agar medium (Biolife Italiana Srl, Milan, Italy) and incubated at 36 ± 1 °C for 48 h. When dark pink-red colonies developed the membrane was transferred to a plate containing Bile Esculin Azide agar medium (Biolife Italiana Srl, Milan, Italy) and incubated at 44 °C for 2 h. Brown colonies with brown-black halos and positive catalysis were identified as Enterococci [15].

### 2.5. P. aeruginosa Investigation

A 250 mL aliquot of melted ice was filtered, the membrane was placed on a plate containing Pseudomonas Selective Agar supplemented with cetrimide (0.20 g) and nalidixic acid (15 mg) (Microbiol, Cagliari, Italy) and incubated at 36 ± 2 °C for 48 h, and green colonies were confirmed to be *P. aeruginosa* [16].

### 2.6. S. aureus Investigation

A 250 mL aliquot of melted ice was filtered and the membrane was placed on a selective, differential ground plate containing Mannitol Salt Agar (Biolife Italiana srl, Milan, Italy). After 48 h of incubation at 36 ± 1 °C, the yellow colonies that were yellow halo-, catalase- and coagulase-positive were identified as *S. aureus* [17].

### 2.7. Fungi Investigation

A 100 mL aliquot of melted ice was filtered and the membrane placed on Sabouraud dextrose agar containing chloramphenicol (0.5 g/L) (Liofilchem, Roseto degli Abruzzi, Italy). After 8 days of incubation at 28 °C, yeast colonies were identified using a semi-automated sugar assimilation system (API ID 32C, Biomerieux, Marcy l’Etoile, France), while filamentous fungi were identified by evaluating their macroscopic and microscopic morphological characteristics according to the methods described elsewhere [18].

### 2.8. Enumeration of Culturable Micro-Organisms

Two 1 mL aliquots of melted ice were separately mixed with yeast extract agar (Biolife Italiana Srl) in Petri dishes, and incubated at 36 ± 2 °C for 48 h and 22 ± 2 °C for 72 h, respectively. The colonies grown in each plate were counted and the results were expressed as the number of colony-forming units per millilitre (cfu/mL).

### 2.9. Statistical Analysis

Because compliance with mandatory or non-mandatory parameters is a qualitative variable, we summarised as a count or a percentage. The chi-square test was employed for comparison between independent groups and, where appropriate, the *p*-values of multiple comparisons were corrected by applying the Bonferroni correction. Bacterial counts are a quantitative variable, but as they were not normally distributed, they are described herein as the median and range. Comparisons between independent groups were conducted with the non-parametric Mann–Whitney U test. *p*-values <0.05 were considered to be statistically significant. Data were analysed with the MedCalc Statistical Software version 19.1.3 (MedCalc Software bv, Ostend, Belgium; https://www.medcalc.org; 2019).

## 3. Results

**Figure 1:**
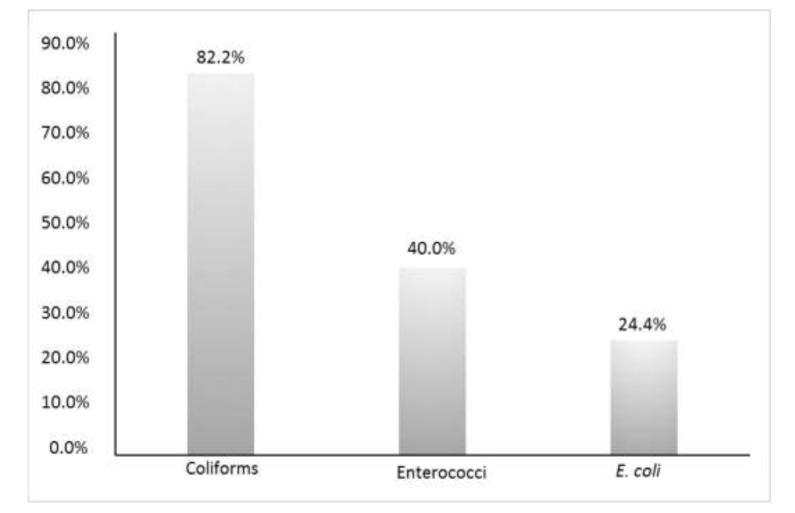
Percentage of non-compliant food ice samples. Samples could be positive to multiple parameters.

According to Decree No. 31/2001, of 99 food ice samples 54 (54.5%) complied with the mandatory parameters. Of these, 52 (96.2%) were positive for additional parameters, in that fungi were isolated from 96.2% of them, *P. aeruginosa* was isolated from 14.8% of them and *S. aureus* from 11.1% of them. The remaining 45 (45.5%) samples were non-compliant for the mandatory parameters (Coliforms 82.2%, Enterococci 40% and *E. coli* 24.4%) (Figure 1). Of these, 43 (95.5%) samples were also positive for the additional parameters in that fungi were isolated from 95.5% of them, *P. aeruginosa* from 40% of them and *S. aureus* from 6.7% of them. The difference between the compliant and non-compliant samples for the additional parameters (96.2% vs. 95.5%, respectively) was not statistically significant (chi-square = 0.37, *p* = 0.5405) (Table 1). After Bonferroni adjustment, the percentage of *P. aeruginosa* showed a statistically significant difference between the compliant and non-compliant groups (*p* = 0.0144), differently from *S. aureus* (*p* = 0.669).

Fungi were identified in 95.8% (95/99) of the samples and no statistically significant difference between the compliant and non-compliant groups was observed after Bonferroni adjustment (*p* = 0.8867) (Table 1). Altogether filamentous fungi and yeasts were detected in 46.3% (44/95) and 20.0% (19/95) of them, respectively, with equal distribution in compliant and non-compliant groups. Mixed fungal species were detected in 32 samples (33.6%). The main filamentous fungi were *Aspergillus* (42.3%), *Penicillium* (17.4%), *Cladosporium* (16%), *Fusarium* (6%), *Paecilomyces* (6%) and *Alternaria* (3%). The following yeasts were also identified: *Candida humicola* (29.4%), *Rhodotorula mucilaginosa* (20%)*, Candida lipolytica* (17.9%), *Candida inconspicua* (12.6%), *Candida intermedia* (8.4%), *Saccharomyces cerevisiae* (6%) and *Candida lusitaniae* (5.2%).

**Figure 2:**
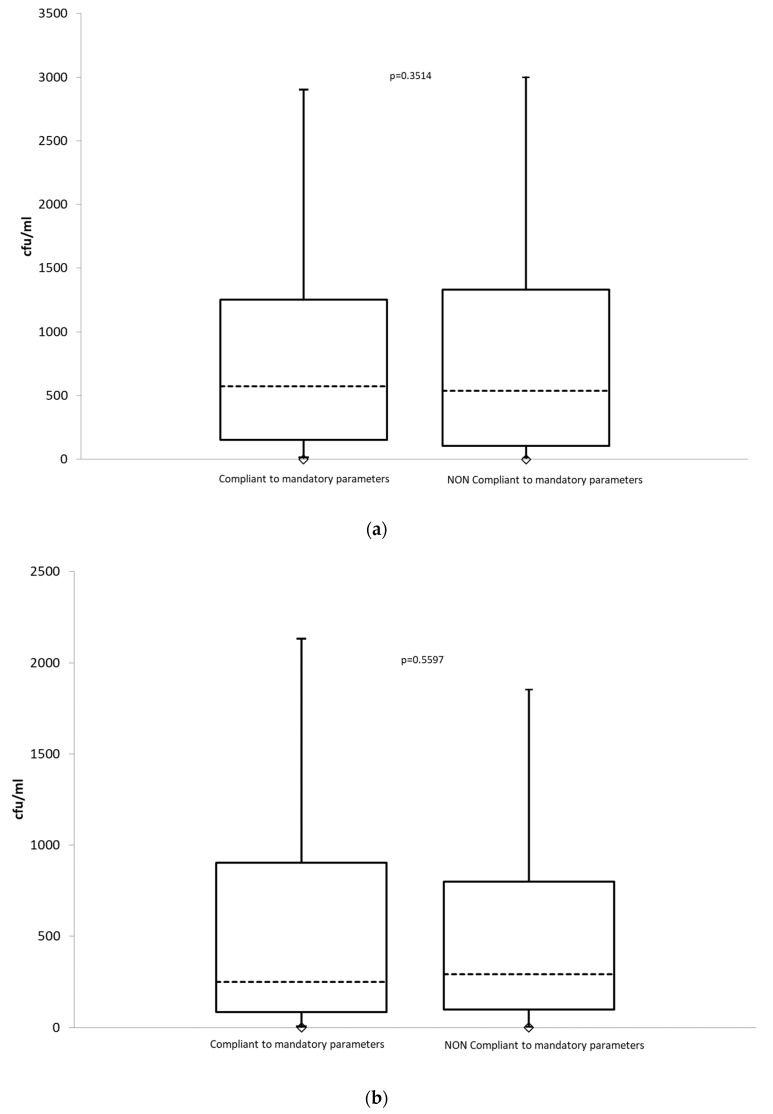
(**a**) Box-plot to compare microbial counts at 22 °C between compliant and non-compliant samples to mandatory parameters. (**b**) Box-plot to compare microbial counts at 37 °C between compliant and non-compliant samples to mandatory parameters.

The median count of mesophilic microbes at 22 °C in the compliant samples was 574 cfu/mL (range 11–3000 cfu/mL), whereas in the non-compliant samples it was 536 cfu/mL (range 11–3000 cfu/mL); a non-statistically significant difference (*p* = 0.3514, Figure 2a) was observed. The median count of the mesophilic microbes at 37 °C in the compliant samples was 292 cfu/mL (range 5–3000 cfu/mL), whereas in the non-compliant samples it was 250 cfu/mL (range 4–3000 cfu/mL); a non-statistically significant difference (*p* = 0.5597, Figure 2b) was observed.

## 4. Discussion

Food ice can be a transmission vehicle for various microorganisms associated with foodborne diseases when hygiene standards for ice production are not respected. Its contamination can occur in successive stages with respect to the water supply, as one or more critical points may be present within the water network. Indeed, the causes of ice contamination, which are manifold, can relate to poor or insufficient quality of drinking water systems, work environments not properly sanitised, ice machines not sufficiently disinfected, insufficient attention to the Hazard Analysis and Critical Control Point (HACCP) principles or to the Manual of Good Hygiene Practice for the production, treatment, storage and use of ice [19].

Some studies have investigated the microbiological quality of food ice, highlighting the presence of bacteria such as *E. coli*, Enterococci, *Pseudomonas* spp., *Acinetobacter* spp., *Stenotrophomonas maltophilia* and *Yersinia* spp. in such ice [20,21,22]. Furthermore, some studies aimed at detecting the presence of yeast and moulds in the ice cubes produced and consumed in Italian bars and restaurants reported that *Candida parapsilosis*, *Cryptococcus* spp. and *Penicillium glabrum* were all able to survive in alcoholic and soft drinks in the presence of contaminated ice, and proposed that Enterococci, *Pantoea conspicua* and *Stenotrophomonas maltophilia* contamination should be reduced in such ice [7].

In developing countries where hygiene and health conditions are considered to not be optimal and cholera is spread in its endemic form, the consumption of food coming into contact with ice was shown to be an important risk to human health. In these geographical areas, the consumption of beverages produced and purchased by street-food and food businesses not constantly subject to specific controls performed by the health authorities contributes to an increasing transmission risk of foodborne diseases [12,23,24].

In Italy, the Ministry of Health has validated and disseminated the Manuals of Good Hygiene Practice delineated by the National Food Ice Institute [2]. They highlight the best operating practices to adopt for the production of food ice and for the drinking water treatment systems installed in public establishments. Both documents provide useful support for carrying out risk assessments correctly so that preventive actions are taken during the ice production phases and critical control points are identified, including physical and mechanical treatments. The same documents focus attention on the usefulness of ordinary and extraordinary maintenance, as well as on the correct sanitisation of the systems, the shortcomings of which certainly contribute to an increased health risk potentially attributable to one or more non-conformities that could make the ice unsuitable for human consumption. The same recommendations were made by the American Centres for Disease Control and Prevention.

In the present study, almost half of the food ice samples we tested were contaminated with *E. coli* and Enterococci (non-compliant samples), although they were derived from potable water. In fact, all ice samples, except four, were positive for *P. aeruginosa, S. aureus* and fungi (additional parameters). Notably, fungi were isolated from approximately 95% of the samples, with filamentous fungi (*Aspergillus, Penicillium, Cladosporium*) being most prevalent. It is important to focus attention on the fact that almost all the samples suitable for the mandatory parameters were positive for other microorganisms that fall within the additional parameters, according to Decree No. 31/2001, probably related to some variables such as water network or unsuitable sanitation of machineries for the production and distribution of ice. Of concern, some microorganisms such as *P. aeruginosa* and *S. aureus* are pathogenic bacteria that are able to cause infections in different bodily sites. Man himself could represent a source of contamination for these microorganisms, especially regarding *S. aureus*; not only its presence among food workers was demonstrated, so too the diffusion of methicillin-resistant *S. aureus* (MRSA) [25]. Thus, contaminated ice can pose a risk in the context of health care-related activities as well, both indirectly (as a potential source of diffusion of microorganisms) [26,27], and directly because ice can be used without suitable sanitary practices in place.

In this regard, some studies have reported on epidemic levels of infection of surgical wounds with *Enterobacter cloacae* during cardiac cardioplegia, and food ice remains the subject of current recommendations for pain relief resulting from mucositis and candidiasis of the oral cavity in patients receiving chemotherapy treatment [28,29]. Therefore, the high isolation of filamentous fungi in our study underlines the risk of infectious complications to which cancer patients are exposed because they use ice cubes to relieve the pain of mucositis resulting from chemo- and radiotherapy treatments. In these cases, the lesions due to mucositis represent an entry route for the fungal spores and therefore a high risk of invasive mycoses, in particular in the neutropenic patient such as paediatric and adult haematological patients or new-borns recovering in intensive care who are exposed to fungal infections of complex therapeutic management [30,31,32,33].

In light of these considerations, it is important that careful monitoring is undertaken to verify compliance with the parameters set out for drinking water in food-producing companies to ensure that they comply with the principles of the HACCP System, including monitoring the sampling checks performed by such companies, as well as drawing up water safety plans, especially for companies that do not benefit from a public water supply.

It is appropriate to remember that under the current legislation, the Services of Prevention Departments in the Local Health Units involved in territorial activities (food hygiene, nutrition services and veterinary services) must carry out annual local planning of the official controls, taking into account the provisions of the Multiannual Control Plan, a national plan aimed at rationalising controls and optimising the use of available resources. Therefore, local planning departments must provide for the use of a “risk-based” method for food ice, taking into account the results of the present investigation, and where no official samples of food ice are normally included it would be appropriate to consider incorporating this matrix into their sampling schemes.

Finally, we highlight that our ice samples were collected in public and collective catering establishments from active ice machines connected to a public water supply network, without highlighting failures or reporting anomalies on plants. It is possible to suppose that public water supply was already contaminated. However, with the Guidelines for Drinking Water Quality [34] of 2004, the World Health Organization has set out the criteria for the Water Safety Plan as the most efficacious means of systematically ensuring the safety of drinking water systems and the quality of the water supplied, protecting the health of consumers [35]. The Italian Guidelines [36] adopted this model, subsequently implemented also in the Apulia region [37], with the objective of integrated risk assessment and management, protecting water supplies from their origin to the point of supply [35]. Thus, it is more probable that contamination is connected with the production and distribution of ice.

Therefore, as regards the equipment, it is strictly necessary to follow the instructions provided by the manufacturer for adequate maintenance; to implement the frequency of proper sanitisation procedures for the internal walls of ice machines, promoting abundant rinsing later; to provide for the replacement of any filters and carry out near-term ice sampling, collected by Food Business Operators, in order to detect any non-compliance early and to promptly take corrective actions. Furthermore, a storage tank, if present, should be subjected to daily checks of hygiene conditions, and its cleaning and descaling should take place at least weekly.

## 5. Conclusions

Our results, although preliminary, have highlighted important sanitation problems in the distribution of ice for use in food and drink. In the face of the evidence shown herein and the desirable planned control actions, which should be adopted by the competent authorities, it seems appropriate to suggest the need for a thorough evaluation of where and when food ice is used, and for the application of the relevant protocols and regulations to ensure proper maintenance and sanitation of ice production plants, as well as timely information and training of staff involved in all aspects of food ice production, storage and use.

In conclusion, further studies need to evaluate the presence of other microorganisms (e.g., pathogenic enterobacteria, enterovirus, protozoa) and to verify a possible relationship between the degree of contamination of the ice and the correct use of the various equipment. Furthermore, research could be extended to other regions of Italy, evaluating also geographic position as a variable and comparing public and collective catering establishments. This would define a more complete and detailed scenario.

## Figures and Tables

**Table 1:** Additional parameters investigated in compliant and non-compliant food ice samples.

	Food Ice Samples		
	No (%)	No (%)	*p*-values
	Compliant 54/99 (54.5)	Non-Compliant 45/99 (45.5)	
Additional parameters	52 (96.2)	43 (95.5)	0.5405
*P.aeruginosa*	8 (14.8)	18 (40)	0.0144 *
*S.aureus*	11 (11.1)	4 (6.7)	0.669 *
Fungi	52 (96.2)	43 (95.5)	0.8867 *
	median (range)	median (range)	
Total microbial count at 22 °C (cfu/mL)	574 (11–3000)	536 (11–3000)	0.3514
Total microbial count at 37 °C (cfu/mL)	292 (5–3000)	250 (4–3000)	0.5597

*: *p*-values after Bonferroni adjustment.
